# Laboratory diagnostics and follow-up of medullary thyroid cancer

**DOI:** 10.1530/JME-25-0185

**Published:** 2026-02-17

**Authors:** Luca Giovanella, Federica D’Aurizio, Petra Petranović Ovčariček, Jacquelien J Hillebrand

**Affiliations:** ^1^ Nuclear Medicine and Thyroid Center, Gruppo Ospedaliero Moncucco, Lugano, Switzerland; ^2^ Nuclear Medicine and Thyroid Center, University Hospital of Zurich, Zurich, Switzerland; ^3^ Clinical Chemistry and Endocrinology, Medysin, Luzern, Switzerland; ^4^ Clinical Pathology, Department of Laboratory Medicine, University Hospital of Udine, Udine, Italy; ^5^ Oncology and Nuclear Medicine, University Hospital Center Sestre Milosrdnice, Zagreb, Croatia; ^6^ School of Medicine, University of Zagreb, Zagreb, Croatia; ^7^ Endocrine Laboratory, Department of Laboratory Medicine, Amsterdam UMC, University of Amsterdam and Vrije Universiteit Amsterdam, Amsterdam, The Netherlands; ^8^ Amsterdam Gastroenterology Endocrinology & Metabolism, Amsterdam, The Netherlands

**Keywords:** medullary thyroid carcinoma, calcitonin, procalcitonin, biomarker standardization, laboratory interference

## Abstract

Medullary thyroid carcinoma (MTC) is a rare neuroendocrine malignancy of thyroid C-cells characterized by the secretion of several circulating biomarkers, including calcitonin (CT), procalcitonin (PCT), carcinoembryonic antigen (CEA), carbohydrate antigen 19-9 (CA 19-9), and pro-gastrin-releasing peptide (proGRP). These analytes substantially contribute to the diagnosis, postoperative monitoring, and prognostic stratification of MTC. Nevertheless, their optimal use remains limited by analytical, pre-analytical, and biological factors that can compromise result reliability and clinical interpretation. Despite improvements in assay technology, significant inter-method variability persists for CT and CA 19-9, while heterophile antibodies, macro-analyte formation, renal dysfunction, and pharmacologic influences may cause spurious or misleading results. Moreover, a lack of harmonized reference intervals and clinical decision thresholds complicates longitudinal follow-up and inter-laboratory comparison. This review systematically addresses current laboratory challenges affecting MTC biomarkers, summarizes the main sources of false-positive or unreliable results, and discusses the complementary diagnostic roles of CT, PCT, and emerging analytes such as proGRP. Emphasis is placed on standardization needs, verification of analytical performance, and the importance of consistent assay use in patient follow-up. Ultimately, the effective management of MTC biomarkers requires active engagement of clinical chemists and pathologists within multidisciplinary teams to ensure accurate interpretation, resolve analytical ambiguities, and integrate biochemical data into evidence-based therapeutic decision-making.

## Introduction

Medullary thyroid carcinoma (MTC) is a rare form of neuroendocrine thyroid cancer originating from the C-cells of the thyroid gland, which produce calcitonin (CT). MTC accounts for approximately 2–5% of all thyroid malignancies, but it is more aggressive compared to well-differentiated thyroid carcinomas. The incidence of MTC is estimated at 0.2–0.3 cases per 100,000/year (1). Hereditary and sporadic forms account for ∼25 and ∼75% of MTC cases, respectively. The clinical, pathological, and molecular distinctions between inherited and sporadic forms are critical for diagnosis and management. The most relevant characteristics are summarized in Table 1. Several serum and tissue biomarkers play a relevant role in the diagnosis, follow-up, and risk stratification of MTC. Calcitonin, procalcitonin, and CEA remain the cornerstone first-line biomarkers: their use has transformed disease management by enabling early detection, postoperative surveillance, and therapeutic monitoring. Serum chromogranin A can also be increased in some patients with MTC, but available evidence indicates that it lacks the diagnostic specificity and sensitivity of calcitonin and CEA and has limited added value in routine MTC diagnosis or management (2). Finally, serum CA 19-9 and proGRP, widely used in the management of pancreatic cancer and small cell lung cancer, are currently being explored as repurposed biomarkers for MTC, particularly for prognostic stratification. However, the measurement and interpretation of these biomarkers remain affected by multiple laboratory-related and biological challenges. These include inter-method variability, pre-analytical instability, and analytical interferences (e.g., heterophile or anti-animal antibodies and macro-analyte formation), as well as biological confounders such as renal dysfunction or pharmacologic effects. Moreover, the lack of harmonized reference intervals and cutoff criteria across different analytical platforms further complicates longitudinal interpretation. This review was therefore prompted to focus on laboratory-related aspects of MTC biomarker testing. It aims to provide a critical overview of pre-analytical, analytical, and interpretative pitfalls affecting MTC biomarkers and to offer practical guidance for both laboratory specialists and clinicians. The ultimate goal is to support the accurate interpretation of biomarker results, minimize diagnostic uncertainty, and strengthen interdisciplinary collaboration in the management of patients with MTC.

**Table 1:** Relevant clinical, pathological, and molecular characteristics of inherited and sporadic MTC.

Feature	Inherited MTC	Sporadic MTC
Prevalence	∼20–25% of all MTC cases	∼75–80% of all MTC cases
Genetic basis	Germline RET proto-oncogene mutation (autosomal dominant)	Somatic RET or RAS mutations
		Germline RET mutations in about 7% of cases
Syndromic association	MEN 2A, MEN 2B, or FMTC	None
Age at onset	Childhood or young adulthood	Typically 5th–6th decade
Sex distribution	No significant differences	Slight female predominance
Foci distribution	Multifocal and bilateral tumor	Unifocal tumor in most cases
CCH	Virtually always present	Usually absent or focal
Associated tumor	MEN2A: pheochromocytoma and HPT	None
	MEN2B: pheochromocytoma, neuromas, and Marfanoid habitus	
RET mutations#	Germline in codons 609, 611, 618, 620 (exon 10), 634 (exon 11) in MEN2A/FMTC; M918T (exon 16) in MEN2B	Somatic M918T. RAS mutations (∼10–20%) when RET-negative. Germline mutations: FMTC/MEN2A-type variants
Serum calcitonin	Markedly elevated	Elevated in proportion to tumor burden
Clinical presentation	Often detected by screening in RET carriers or as MEN-related symptoms	Solitary thyroid nodule; may present with neck mass or metastatic disease
Family history	Positive in > 90%	Usually negative
Prognosis	Variable; depends on RET codon and timing of prophylactic surgery	Worse when detected later
Management	Genetic counseling, RET testing, and prophylactic thyroidectomy in carriers	Managed as sporadic malignancy; germline RET mutation screening currently indicated

MTC, medullary thyroid carcinoma; RET, rearranged during transfection; RAS, rat sarcoma; MEN, multiple endocrine neoplasia; FMTC, familial MTC; CCH, C-cell hyperplasia; HPT, hyperparathyroidism; #most frequent mutations are reported.

### Diagnosis and treatment of MTC: basic concepts

The diagnostic flow chart of MTC typically begins with the detection of a thyroid nodule by ultrasound or clinical examination. When fine-needle aspiration cytology (FNAC) suggests MTC or shows indeterminate cytology, serum CT measurement is obtained. Elevated CT levels (i.e., >100 pg/mL) strongly support MTC and prompt locoregional and distant staging. Adding CT measurement on FNAC washout fluids proved to be highly accurate to confirm/exclude the presence of MTC, especially in cases with indeterminate serum CT levels (i.e., 10–100 pg/mL). More recently, it has been shown that the add-on measurement of serum procalcitonin (PCT) in such cases provides a quite absolute negative predictive value without the need of an additional FNA procedure (see below). The clinical course ranges from indolent to aggressive, and early total thyroidectomy with routine central compartment dissection offers the best chance of cure.

### Biomarkers used in monitoring medullary thyroid carcinoma

Serum calcitonin (CT) and carcinoembryonic antigen (CEA) measurements, together with their doubling-time (DT) calculations, are central components of postoperative monitoring in MTC. These biomarkers are routinely integrated with periodic neck ultrasound and, when indicated by rising marker levels or shortening DT, second-level imaging modalities, such as computed tomography, magnetic resonance imaging, and positron emission tomography. In patients with relapsing or advanced disease, locoregional control is achieved through surgery and external-beam radiation therapy, whereas systemic treatment options include multikinase inhibitors (e.g., vandetanib and cabozantinib) and selective RET inhibitors (e.g., selpercatinib and pralsetinib). Conventional chemotherapy has a limited role and is generally reserved for rapidly progressive or symptomatic disease that is refractory to targeted therapies (3). Although circulating biomarkers represent essential tools for the diagnosis, follow-up, and management of MTC, their clinical interpretation may be affected by pre-analytical, analytical, and post-analytical factors. The following sections review the currently available biomarkers used in MTC monitoring, with particular emphasis on these limitations and on emerging strategies aimed at improving their diagnostic and prognostic performance.

## Calcitonin

Calcitonin (CT) was first described in the early 1960s as a peptide hormone secreted by the C-cells in the thyroid gland (4). CT is derived from pre-prohormone pre-PCT (141 amino acids). Endopeptidase activity and posttranslational processing render PCT (116 amino acids) and, consequently, CT (32 amino acids). The most prominent physiological role of CT is believed to be in maintaining calcium homeostasis via inhibiting osteoclast activity and renal calcium reabsorption (5). However, patients with absent CT following total thyroidectomy do not manifest disturbed calcium homeostasis, suggesting a degree of redundancy (6).

### Analytical and pre-analytical considerations

The first immunoassay for measuring CT in human thyroid tissue extracts was described in 1968 (7).

The initial radioimmunoassay (RIA) was followed up by more sensitive and specific immunometric assays, finally using monoclonal antibodies and implementing (electro)chemiluminescence technology on automated platforms, which further improved the analytical sensitivity and specificity for monomeric CT, minimizing cross-reactivity with CT precursors (PCT) or CT gene-related peptides (8). The analytical sensitivity of the current automated CT immunoassays is as low as 0.5 pg/mL, making it possible to detect small elevations from basal CT levels. Calcitonin measurement requires strict pre-analytical standardization. Blood should be collected in validated serum or EDTA plasma tubes, with consistent use of the same matrix for follow-up. Samples must be centrifuged and separated within 1–2 h after collection. If analysis is delayed, samples should be stored at 2–8°C for short-term or frozen (≤−20°C, preferably −70/−80°C) for longer-term storage, avoiding repeated freeze–thaw cycles. Transport should ensure appropriate temperature control. Samples with unverified or inadequate pre-analytical conditions should be considered non-conforming, and results should not be reported or must be clearly qualified, with repeat sampling recommended. Although all current immunoassays are traceable to the World Health Organization International Standard 89/620, standardization issues for different CT assays are still present. Interchangeability of CT measurements from different immunoassays is therefore still hampered. Kahaly *et al.* showed that the Siemens Immulite 2000 assay measured on average 20% higher than the Roche Elecsys assay (9), whereas Mater *et al.* showed that the Siemens Atellica IM 1300 assay measured 13% higher than both Roche Elecsys and Diasorin Liaison XL assays (10). In individual cases, differences between immunoassays may be even greater, possibly due to CT circulating in multiple isoforms or fragments that are not recognized by all immunoassays. The poor interchangeability of CT measurements argues for consistent use of a particular immunoassay for serial monitoring during patient follow-up. CT immunoassays are, like other (MTC biomarker) immunoassays, prone to analytical interferences. For instance, the presence of heterophilic antibodies, specific human-anti-mouse antibodies (HAMA), or human-anti-human antibodies, like rheumatoid factor, in sera may result in falsely high or low CT levels. If CT levels are unexpectedly high or low in relation to the clinical context, it is recommended to measure CT levels using another CT immunoassay (using different antibodies) or pretreat samples to block interfering antibodies (8). In cases with inconsistently high CT levels, pretreating samples with polyethylene glycol can be useful, as falsely elevated calcitonin levels may result from immunoreactive high-molecular-mass complexes of calcitonin bound to immunoglobulins (macrocalcitonin). Polyethylene glycol precipitation leads to removal of the calcitonin–immunoglobulin aggregates, resulting in low calcitonin recovery in the measured supernatant and thereby helping to identify this analytical interference (11). Conversely, the analytical high-dose hook effect may result in falsely low CT measurements in patients with extremely high CT levels. In such cases, CT occupies both the capture as the detection antibody, thereby preventing the formation of the antibody–antigen–antibody complex and resulting in falsely low CT measurements. This may particularly occur in one-step immunoassays without intermediate washing steps. Running high dilutions easily unmasks the high-dose hook effect. In addition, interferences with streptavidin–biotin complexing (e.g., anti-streptavidin, anti-biotin, and biotin) or detection tags (e.g., anti-ruthenium) may occur (assay-dependent). Pre-analytical factors that can influence CT measurements include prolonged processing of blood samples and inadequate storage. Serum proteases are thought to rapidly degrade CT in blood, necessitating rapid sample processing and freezing. Few stability studies have been performed; most studies for practical reasons have been performed in sera and not whole blood, and the interpretation of the findings from different studies is complicated due to differences in assays used and experimental setups (pooled sera vs individual sera, spiked or naive sera) (12). Mater *et al.* have recently demonstrated that following rapid processing, serum CT remained relatively stable for up to five hours at room temperature (10). In addition, repetitively freezing and thawing (up to five times) serum only had a marginal impact, if any, on CT levels in serum. Other factors that may affect the pre-analytical process include physiological conditions, such as sex and age (see below), and the use of drugs that interfere with the H+/K+ ATPase proton pump (which increase gastrin and, ultimately, CT levels). Smoking has been associated with mildly increased serum calcitonin (CT) levels – particularly in men – likely reflecting secretion from pulmonary neuroendocrine cells rather than thyroidal C-cells (13). More broadly, elevated CT is not specific for MTC and may also be observed in benign conditions, such as C-cell hyperplasia, renal dysfunctions, infections/inflammatory diseases, and hypergastrinemia (most frequently, secondary to proton pump inhibitors) (12, 14). Autoimmune thyroiditis has long been considered a potential cause of increased calcitonin levels; however, this association has recently been re-examined, and current evidence suggests that the observed elevations may be more closely related to changes in thyroid volume rather than to autoimmunity ‘per sè’ (see below) (15). Finally, aspecific increases in CT can be observed non-MTC malignant conditions, such as neuroendocrine neoplasms, leukemia, small cell lung cancer, breast cancer, and pancreatic cancer (16). Therefore, serum calcitonin should be interpreted with caution, as mild to moderate elevations may be related to smoking-associated pulmonary neuroendocrine secretion or different benign conditions, while non-specific increases have also been described in non-MTC malignancies, particularly neuroendocrine neoplasms. Table 2 summarizes the principal benign and malignant non-MTC causes of increased serum calcitonin and their typical magnitude of elevation.

**Table 2:** Benign (d’Herbomez *et al.* (14); Kratzsch *et al.* (12)) and malignant (Algeciras-Schimnich *et al.* (16)) causes of serum calcitonin elevation in conditions other than MTC.

Cause	Typical range (pg/mL)	Comments
Benign conditions		
Smoking	10–15	Usually reversible
Increased thyroid mass (e.g., goiter)	10–20	Increased number of C-cells
Autoimmune thyroiditis	10–15	Higher thyroid volume
Proton pump inhibitor use/hypergastrinemia	10–20	Normalize after withdrawal
Chronic kidney disease (mild–moderate)	10–20	Reduced clearance
Chronic kidney disease (advanced)	20–50	Rarely > 100
C-cell hyperplasia (sporadic)	20–50	Common cause in gray zone
Severe C-cell hyperplasia	50–100	Rare outside multiple endocrine neoplasms
Autoimmune gastritis/pernicious anemia	15–40	Via hypergastrinemia
Hypercalcemia (non-MTC)	10–20	Calcium-stimulated secretion
Acute systemic inflammation/sepsis	10–40	Transient
Interference (heterophile Ab, macro-CT)	Any	Suspect if discordant
Physiological (children)	10–20	Age-related
Malignant diseases		
Small cell lung cancer	100–1,000+	Ectopic calcitonin secretion
Bronchial neuroendocrine tumor	50–300	Thyroid imaging negative
Pancreatic neuroendocrine tumor	20–300	Variable secretion
Gastrointestinal neuroendocrine tumor	20–100	Usually lower than MTC
Pheochromocytoma/paraganglioma	20–80	Rare
Prostate neuroendocrine carcinoma	50–300	Advanced disease
Breast carcinoma (neuroendocrine features)	20–80	Rare
Ovarian neuroendocrine carcinoma	50–300	Rare, aggressive
Differentiated thyroid carcinoma	10–20	Indirect C-cell stimulation

MTC, medullary thyroid carcinoma.

### Reference ranges and cutoff levels

Basal CT levels in healthy individuals are typically below 10 pg/mL. However, reference ranges for CT are immunoassay and population dependent and should ideally be determined in each separate laboratory. A positive correlation between calcitonin levels and thyroid volume has been demonstrated in both animal models and in humans (17, 18). Different studies consistently showed higher CT levels in men than in women, which may be explained by a higher thyroid mass and C-cell number in men than in women (12, 14, 18). Others also showed age-dependency of CT levels, with children having higher CT levels, gradually decreasing upon aging toward adolescence. This suggests that not only sex-specific but also age-specific reference ranges should be used (14). Basal CT levels above 100 pg/mL are indicative of MTC, but levels between 10 and 100 pg/mL are typically considered in the gray zone and are more frequently not attributed to MTC but C-cell hyperplasia (19). Importantly, these cutoff levels are only indicative, as they are assay-dependent. It would be advisable to include cutoff levels based on fold-increase from local reference ranges rather than (generalized) absolute CT levels. Several groups have emphasized that slightly elevated basal calcitonin values in the diagnostic ‘gray area’ should not be interpreted as a single static result, but rather in the context of their kinetics over time, especially in patients with thyroid nodules, where non-specific causes of hypercalcitoninemia are possible. Reflecting this concept, German recommendations and the updated German S3-oriented guidance adopt sex-specific action thresholds (≈30 pg/mL in women and 60 pg/mL in men) to balance the risk of unnecessary thyroidectomy against the risk of missing early micro-MTC. In patients with nodules and basal CT values below these intervention thresholds, a repeat-measurement strategy (every 3–6 months) is proposed, with surgery primarily triggered by a clear upward trend or threshold crossing, thereby leveraging ‘dynamics’ as a pragmatic discriminator between indolent/non-specific elevations and evolving MTC (20). This approach is further supported by outcome-oriented observations that early-stage disease associated with relatively low calcitonin concentrations remains highly curable, so short-term surveillance within the gray area can preserve excellent prognosis while improving the reliability of basal calcitonin-based screening pathways.

### Clinical utility

There is no general agreement over whether or not to measure serum CT levels for screening for MTC. For sporadic MTC, the European Thyroid Association guidelines recommend measuring CT, but considering the low prevalence, suggest selecting patients with thyroid nodules and scheduled for surgery, those with nodules with indeterminate cytology, or suspicious findings on ultrasound (21). The American Thyroid Association, however, does not give tailored advice on whether or not to measure serum CT in patients presenting with thyroid nodules (22). Consensus has been achieved on performing CT measurements for screening patients with a family history of MTC or MEN type 2A and 2B and to guide the timing of prophylactic thyroidectomy in those with genetic MTC (22). Notably, MTC rarely presents without elevated serum CT levels (1–2% of cases), but if so, this could be explained by, i.e., impaired secretion, CT isoforms, or analytical issues as described above. In such cases, measurement of other biomarkers for MTC may be particularly helpful (8, 23).

### Post-surgical monitoring

Following a total thyroidectomy, CT levels in serum decline and normalize within days to weeks. During postsurgical monitoring, the definition of biochemical remission based on calcitonin (CT) levels should take into account the analytical characteristics of the assay used. In particular, a distinction should be made between CT concentrations below the limit of quantification (LoQ) and values falling within the reference range of thyroid-healthy individuals. When highly selective assays targeting the monomeric (mature) form of calcitonin are used, postoperative CT levels below the LoQ represent the most stringent criterion for biochemical cure (24). Conversely, with less selective assays, postoperative CT values within the reference range are also considered consistent with biochemical remission (25). Importantly, clinical outcomes are generally favorable even in patients with slightly detectable or mildly elevated postoperative CT levels, indicating that the absence of a strict biochemical cure does not necessarily translate into an adverse prognosis. Conversely, rising CT levels following total thyroidectomy suggest residual disease or recurrence. The CT doubling time (DT) can be used to assess tumor burden and the aggressiveness of MTC. This dynamic parameter captures tumor kinetics and can provide valuable prognostic and monitoring information. At least ≥3–4 sequential CT results are needed (the same assay) during post-surgical monitoring. Validated log-linear DT calculators are available and employed in clinical practice (i.e., https://thyroid.org/professionals/calculators/, https://www.guidelinecentral.com/calculator/408/ or https://en.kuma-h.or.jp/tools). In postoperative patients, rising marker kinetics with DT shorter than 6–24 months indicate a high risk of structural recurrence and poor prognosis. Intermediate DT values suggest a moderate risk, whereas a DT longer than two years generally reflects a more indolent course and allows for less intensive follow-up.

### Calcitonin stimulation test: is it still a useful procedure?

In the past, stimulation with pentagastrin was performed to distinguish between individuals with slightly elevated (gray zone) basal CT levels due to non-MTC and those with MTC. Pentagastrin-stimulated CT was also used during post-surgical monitoring to determine whether residual tissue was present and whether further interventions were necessary. As pentagastrin is no longer available, intravenous calcium infusion (calcium gluconate and calcium chloride) is considered a cheap and relatively safe alternative (26). However, the analytical performance of the current CT assays and the option to measure PCT as a reflex test (see below) reduced the diagnostic need for stimulation testing with the notable exception of risk stratification in RET mutation carriers (22). Additional clinical scenarios remain, in which calcium stimulation may still provide useful diagnostic information. In particular, stimulation testing has been discussed as an aid in the differential diagnosis of elevated basal CT levels when MTC must be distinguished from ectopic calcitonin secretion by other neuroendocrine neoplasms, which typically show a blunted or absent stimulatory response (27). Moreover, calcium stimulation may help clarify the interpretation of increased CT concentrations in patients with advanced chronic kidney disease, especially those undergoing hemodialysis, in whom basal CT levels are frequently elevated in the absence of MTC (28). These selected indications highlight that, despite its declining role, stimulation testing may still be valuable in specific and diagnostically challenging contexts.

## Procalcitonin

Procalcitonin (PCT), the 116-amino acid prohormone of CT encoded by *CALC-1*, is physiologically produced by thyroid C-cells and rapidly cleaved to CT, yielding low serum levels <0.05 ng/mL. During bacterial infection or inflammation, extra-thyroidal tissues markedly upregulate PCT synthesis and, accordingly, PCT was primarily established as a sepsis biomarker (29). Interestingly, PCT may also be increased in patients with MTC, reflecting its parallel synthesis and secretion with calcitonin by neoplastic C-cells. In this setting, PCT concentrations are often persistently elevated and correlate with tumor burden and disease activity rather than with infectious or inflammatory processes.

### Analytical and pre-analytical considerations

Modern automated platforms (e.g., Thermo Fisher (B·R·A·H·M·S) Kryptor, Roche Elecsys, Siemens Atellica, and Diasorin Liaison) employ B·R·A·H·M·S PCT immunometric immunoassays. Comparative studies confirmed robust inter-method correlations attributable to shared proprietary monoclonal antibody pairs and reference standards ensuring uniform epitope recognition and calibration traceability. Typical PCT limits of quantification range from 0.02 to 0.05 ng/mL, with within-run coefficients of variation (CV) < 5%. Blood should be collected in validated serum or plasma tubes according to the assay manufacturer’s instructions, with consistent matrix use for serial measurements. Samples should be centrifuged and separated within 2 h after collection. For short delays (≤24 h) before analysis, samples may be stored at 2–8°C; longer storage requires freezing (≤−20°C), avoiding repeated freeze–thaw cycles. Transport conditions must ensure temperature stability. Hemolysis, lipemia, or excessive heat may induce minor interference, while differences between measurements in serum and plasma are negligible. Samples with inadequate or unverifiable pre-analytical conditions should be considered non-conforming, and results should not be reported or must be clearly qualified, with repeat sampling recommended. Overall, PCT demonstrates greater pre-analytical robustness and better standardization, precision, and reproducibility than CT. In contrast to bacterial sepsis, non-infectious causes of procalcitonin elevation typically result in mild to moderate increases, although markedly higher values may be observed transiently after major tissue injury or shock (29). Increased PCT levels have also been reported in other malignancies with neuroendocrine differentiation, including small cell lung cancer and selected gastroenteropancreatic neuroendocrine neoplasms (16). These non-infectious oncologic causes should therefore be considered when interpreting unexplained PCT elevations in the absence of clinical evidence of infection. Relevant causes of serum PCT increase outside MTC are summarized in Table 3.

**Table 3:** Benign (Becker *et al.* (29)) and malignant (Algeciras-Schimnich *et al.* (16)) causes of serum procalcitonin elevation in conditions other than MTC.

Cause	Typical range (ng/mL)	Comments
Benign conditions		
Sepsis, burns, major trauma, and major surgery	2–100+	High in early phase; decreases with recovery
Cardiogenic shock	1–10	Related to tissue hypoperfusion
End-stage renal disease	0.5–5	Reduced clearance
Non-bacterial infections (e.g., viral infections)	0.5–2	Transient
Extracorporeal circulation	0.5–5	Transient, inflammation-related
Liver failure	0.5–3	Limited data
Assay interference	Any (usually < 10)	Rare; suspect when clinical context is discordant
Malignant diseases		
Small cell lung cancer	0.5–20	Rare; usually persistent
Neuroendocrine tumors	0.5–20	Rare; usually persistent

MTC, medullary thyroid carcinoma.

### Reference ranges and cutoff levels

In healthy individuals, serum PCT levels remain <0.05 ng/mL. PCT shows negligible age- or sex-related variations and a predictable, concentration-independent, *in vivo* half-life (∼20–24 h), providing additional analytical stability.

In MTC, PCT levels rise proportionally to tumor burden and parallel CT levels. Cutoff levels >0.1–0.2 ng/mL suggest MTC in the absence of infection, and vice versa, undetectable PCT levels exclude MTC with a high negative predictive value (8).

### Clinical utility

PCT was first proposed as an MTC biomarker by Algeciras-Schimnich *et al.* (16), and subsequent multiple studies confirmed its diagnostic and prognostic value. Across studies, PCT demonstrates a strong correlation with CT (*r* ≈ 0.88–0.96). Cutoff levels between 0.08 and 0.25 ng/mL best discriminate active or structural disease. The marker provides an excellent specificity (≈90–99%) and a negative predictive value (≈100%), particularly valuable for excluding MTC in patients with thyroid nodules and confirming remission during follow-up. Giovanella and colleagues systematically reviewed and meta-analyzed 11 studies (total *n* = 5,817, including 335 MTC cases) to assess the diagnostic and monitoring accuracy of PCT in MTC. They reported a pooled sensitivity of ∼0.90 (95% CI: 0.71–0.97) and specificity of ∼1.00 (95% CI: 0.85–1.00) for PCT, along with a high positive predictive value (∼99%) and high negative predictive value (∼97%) in the studied cohorts including patients at diagnosis and operated MTC patients (30). The high specificity and positive predictive value suggest that PCT is unlikely to yield false positives in non-MTC contexts, which is valuable in clinical decision-making, especially when CT results are equivocal. The favorable (pre-)analytical properties of PCT assays (stability and standardization) complement this diagnostic performance and strengthen the proposition that PCT might function as an alternative to CT (31). However, the established role of CT in clinical practice, the current lack of regulatory approval of PCT for thyroid applications due to the rarity of MTC, and the cost of label expansion limit its widespread adoption instead of CT. Nevertheless, PCT represents a valuable complementary marker to CT, particularly useful when CT levels fall in the gray zone or when assay reliability is of concern (32) (Table 4).

**Table 4:** Procalcitonin in medullary thyroid cancer: clinical utility, (dis)advantages, and comparison with calcitonin.

Diagnosis	Similar sensitivity/specificity for MTC as CT but better NPV. Accordingly, PCT is especially indicated as a rule-out test in patients with calcitonin levels within the gray zone
Follow-up	Similar accuracy for detection of residual/relapsing MTC as CT. PCT is especially indicated in patients with inconsistent CT levels during follow-up (i.e., interferences)
Advantages	Greater assay stability, less susceptible to pre-analytical issues, and better standardized compared to CT
Disadvantages	Out-of-label use in MTC (reimbursement issues), not (yet) an established marker for MTC in guidelines (clinicians are well accustomed to CT use and interpretation, which may partly explain the reluctance to adopt alternative markers)

PCT, procalcitonin; CT, calcitonin; and NPV, negative predictive value.

## Carcinoembryonic antigen

Carcinoembryonic antigen (CEA) was first discovered in 1965 as a glycoprotein antigen present in human embryonic and fetal digestive tract tissues (33). CEA is a heavily glycosylated protein with a molecular weight of approximately 180–200 kDa and belongs to the carcinoembryonic antigen-related cell adhesion molecule family, which is part of the immunoglobulin superfamily. The protein consists of one N-terminal variable immunoglobulin-like domain and six constant immunoglobulin-like domains, with multiple glycosylation sites that contribute to its heterogeneity. It was initially identified in colorectal cancer, but its role has expanded as its elevation has also been detected in MTC. In such a context, CEA is produced by the same C-cells that secrete CT.

### Pre-analytical and analytical considerations

Unlike CT, CEA immunoassays are generally better standardized. Furthermore, CEA is relatively stable in serum samples, which makes it practical for routine clinical use. Blood should be collected in validated serum or plasma tubes according to the assay manufacturer’s instructions, with consistent matrix use for longitudinal monitoring. Samples should be centrifuged and separated from cells within 1–2 h after collection. For short delays (≤24 h) before analysis, samples may be stored at 2–8°C; for longer storage, serum or plasma should be aliquoted and frozen (≤−20°C), avoiding repeated freeze–thaw cycles. Transport must ensure appropriate temperature control. CEA is analytically stable compared with peptide hormones; however, samples with inadequate or unverifiable pre-analytical conditions should be considered non-conforming, and results should not be reported or must be clearly qualified, with repeat sampling under standardized conditions recommended. Serum concentrations may be influenced by paraphysiological factors, such as smoking, age, and sex, and particularly, the biomarker CEA can be produced and released by various cell types and is elevated in numerous benign and malignant pathological conditions, making it less specific as a diagnostic marker for MTC (34, 35, 36). Generally speaking, serum CEA values below 10 ng/mL are most frequently associated with paraphysiological or benign conditions, whereas persistent concentrations exceeding 20–30 ng/mL should prompt evaluation for an underlying malignancy in the appropriate clinical context. Benign and malignant causes of serum CEA increase outside MTC are summarized in Table 5.

**Table 5:** Benign (Trapé *et al.* (35)) and malignant (Goldstein & Mitchell (34)) causes of serum carcinoembryonic antigen (CEA) elevation in conditions other than MTC.

Condition	Typical CEA range (ng/mL)	Remarks
Benign conditions		
Chronic liver disease (cirrhosis and hepatitis)	5–20	Reduced hepatic clearance
Cholestasis/biliary obstruction	10–40	Decreases after relief of obstruction
Inflammatory bowel disease	5–20	Parallels disease activity
Chronic lung disease (COPD and ILD)	3–10	More evident in smokers
Pancreatitis (acute or chronic)	5–15	Usually transient
Renal insufficiency (non-end stage)	5–10	Modest elevation (reduced clearance)
Benign breast disease	5–10	Especially fibrocystic changes
Diabetes mellitus	5–10	Mild, non-specific elevation
Severe infection/systemic inflammation	5–15	Transient increase
Malignant conditions		
Colorectal cancer	5–100+	Correlates with stage and metastases
Pancreatic cancer	10–100+	Sometimes markedly elevated
Gastric cancer	5–50	Variable sensitivity
Lung cancer (adenocarcinoma)	5–50	Correlates with stage and metastases
Breast cancer	5–50	More frequent in advanced disease
Ovarian cancer (mucinous subtype)	5–50	Limited sensitivity
Neuroendocrine neoplasms	5–50	Non-specific; persistent elevations
Leukemia	5–30	Rare, paraneoplastic elevation

MTC, medullary thyroid carcinoma.

### Reference ranges and cutoff levels

In healthy individuals, serum CEA levels remain below 5 ng/mL; for smokers, the upper limit of the reference range is typically 10 ng/mLA. A recent study revealed interesting findings regarding CEA distribution across different patient groups. The median (range) CEA levels in patients with benign thyroid disease, thyroid carcinoma (non-MTC), MTC with evidence of disease, and MTC with no evidence of disease were 1.9 (0.7–938.4), 1.7 (0.3–17.3), 24.6 (1.1–430.3), and 1.7 (1.1–48.9) ng/mL respectively. Notably, the only outlier in the benign group with a CEA level of 938.4 ng/mL was also diagnosed with a metastatic adenocarcinoma of unknown primary origin, demonstrating the non-specific nature of CEA elevation (31). The cutoff level for CEA that is generally used in clinical practice is 5 ng/mL. It represents a balance between sensitivity and specificity, although optimal cutoff levels vary depending on the specific assay and the patient population.

### Clinical utility

The use of CEA in early diagnosis is of no added value since it is not a specific marker for MTC. Schonebaum *et al.* have recently evaluated the diagnostic performance of CEA alone and CEA combined with CT for MTC. The combination of CEA and CT demonstrated a sensitivity of 87.1%, specificity of 100.0%, PPV of 100.0%, and NPV of 98.7%, compared to 87.5% (sensitivity), 92.4% (specificity), 54.9 (PPV), and 98.6% (NPV) for CEA alone, respectively, but did not surpass the performance of the combination of CT and PCT (see above), indicating the primary role of CT (and PCT)-based strategies in MTC diagnosis (31). The primary clinical value of CEA in MTC is prognostic and in disease surveillance (37). Serum CEA levels positively correlate with tumor burden and disease stage. Machens *et al.* found that elevated preoperative CEA levels correlated with the larger size of the primary tumor, regional lymph node involvement, and distant metastases, revealing an even dose-dependent, almost linear relationship between total tumor bulk and increasing CEA levels (38). CEA is also valuable for treatment response evaluation, detection of recurrence, and prognosis assessment. Following complete surgical resection, the persistence of increased CEA levels usually indicates residual tumor tissue, while rising levels suggest disease progression or recurrence. In turn, persistently increased or rising CEA levels are associated with a poorer prognosis (36). Notably, the longer and highly variable half-life of CEA (usually 3–5 days, but may be up to 88 days) compared to CT (a rapid component, half-life of about 3 h, and a slower component, half-life of about 30 h) may sometimes require a longer waiting time postoperatively for the measurement of CEA, compared with CT (2). Progression rates predicted by CEA doubling time (DT) correlate with the progression rates predicted by CT DT. However, while CT DT has a better prognostic value than CEA DT in discordant cases, a progressive increase in CEA levels with stable or declining CT levels indicates disease progression, dedifferentiation, and a worse prognosis (22). All in all, CEA remains an important biomarker in the management of MTC, although its role is primarily complementary rather than primary. Current clinical practice guidelines recommend CEA measurements at initial MTC diagnosis for prognostic assessment and during post-treatment monitoring (combined with CT) as a marker for disease progression, taking into account CEA trends (DT) rather than absolute levels for clinical decision-making.

## Carbohydrate antigen 19-9 (CA 19-9)

Carbohydrate antigen 19-9 (CA 19-9), also known as the sialyl-Lewis A antigen, is a carbohydrate epitope that is typically expressed on glycoproteins and glycolipids, including mucins. It was discovered in the late 1970s and first identified in the serum of colorectal cancer patients (39). Subsequently, elevated CA 19-9 levels were found in pancreatic cancer patients, and very soon, this marker became one of the most used in the clinical management of ductal carcinoma of the pancreas, for both therapeutic response monitoring and prognosis (40). Recently, CA 19-9 has emerged as a potential prognostic marker in MTC: elevated CA 19-9 levels predict more aggressive forms of neoplasia and a significantly shorter overall survival, regardless of serum CT levels (41). The mechanism responsible for the expression of CA 19-9 in advanced MTC remains unclear, and the current literature on the subject is scarce. One hypothesis suggests that, in advanced neoplasia, cell clones resistant to hypoxia may acquire the ability to express markers not typically associated with the tumor’s cell of origin, such as CA 19-9. Unlike CT, which is relatively specific for C-cells, CA 19-9 can be produced by various cell types, and its serum levels are elevated in numerous benign pathological conditions, making this marker not specific for the diagnosis and follow-up of MTC.

### Pre-analytical and analytical considerations

Carbohydrate antigen 19-9 (CA 19-9) measurement requires standardized pre-analytical handling. Blood should be collected in validated serum or plasma tubes according to the assay manufacturer’s instructions, with consistent matrix use for longitudinal monitoring. Samples should be centrifuged and separated from cells within 1–2 h after collection. For short delays (≤24 h) before analysis, samples may be stored at 2–8°C; for longer storage, serum or plasma should be aliquoted and frozen (≤−20°C), avoiding repeated freeze–thaw cycles. Transport must ensure appropriate temperature control. CA 19-9 is analytically stable; however, samples with inadequate or unverifiable pre-analytical conditions should be considered non-conforming, and results should not be reported or must be clearly qualified, with repeat sampling under standardized conditions recommended. Currently, most of the immunoassays for the quantitative determination of CA 19-9 use an immunometric immunoassay format and rely on the monoclonal antibody 1116-NS-19-9 (Centocor). This antibody specifically targets the sialyl-Lewis A glycan epitope, a member of the Lewis blood group antigen family, which is involved in the recognition and binding of glycans, lipids, and proteins. Despite extensive research and widespread clinical use, considerable challenges remain in achieving standardization across CA 19-9 analysis platforms. Automation of immunoassays significantly improved accuracy and operational efficiency but did not eliminate the inter-method variability observed when analyzing identical samples with different analytical systems. Kremser *et al.* have conducted a longitudinal analysis of EQA data for various tumor markers, including CA 19-9, to evaluate both intra- and inter-method performance between laboratories using the most common analytical platforms (42). Their results revealed that intra-assay variability improved considerably, compared to the past, with coefficients of variation (CV) falling below 16% in individual test systems. However, inter-assay variability remained significant and clinically relevant. The inter-assay variability is attributable to the absence of a true reference method and hence several analytical factors, including differences in monoclonal antibody specificity (i.e., distinct epitope recognition), structural heterogeneity of the sialyl-Lewis A antigen, and variations in assay architecture and reagent composition (43). CA 19-9 is also a non-specific biomarker. Besides pancreatic and gastrointestinal neoplasms and MTC, increased serum CA 19-9 levels may be noted in different benign (35) and malignant (44) conditions that may coexist and confound clinical interpretation (Table 6). Thus, any increase in CA 19-9 should be interpreted with caution, especially in the context of comorbidities (8).

**Table 6:** Benign (Trapé *et al.* (35)) and malignant (Maestranzi *et al.* (44)) causes of serum carbohydrate antigen 19-9 (CA 19-9) elevation in conditions other than MTC.

Condition	Typical CA 19-9 range (U/mL)	Remarks
Benign conditions		
Cholestasis/biliary obstruction	100–1,000+	Frequent benign causes; falls after drainage
Acute or chronic pancreatitis	50–500	Transient; overlaps with pancreatic cancer
Chronic liver disease	50–200	Correlates with cholestasis severity
Cholecystitis/cholangitis	100–500	Often inflammatory-driven
Cystic fibrosis	50–500	Chronic pancreatic involvement
Interstitial lung disease	37–100	Rare; mechanism unclear
Renal insufficiency	37–100	Reduced clearance
Malignant diseases		
Pancreatic adenocarcinoma	100–10,000+	Primary indication, correlates with stage
Cholangiocarcinoma	100–10,000+	Often overlaps with benign cholestasis
Gallbladder cancer	100–5,000+	Advanced disease
Gastric cancer	50–1,000	Limited sensitivity
Colorectal cancer	50–500	Usually advanced disease
Ovarian cancer (mucinous)	50–1,000	Non-specific
Hepatocellular carcinoma	50–500	Often with liver dysfunction
Lung cancer (adenocarcinoma)	37–200	Rare, non-specific

MTC, medullary thyroid carcinoma.

### Reference ranges and cutoff levels

In healthy individuals, serum CA 19-9 levels are low. Reference ranges are assay-dependent. Moreover, CA 19-9 expression requires the Lewis gene product, 1,4-fucosyltransferase, present only in individuals with the blood groups Le (ab+) or Le (a+b) (45). Approximately 6 and 22% of the Caucasian and non-Caucasian population are genotypically Le (a−b−) and thus unable to express the sialyl-Lewis A antigen. As a result, these individuals typically exhibit undetectable or very low serum CA 19-9 levels regardless of tumor burden, leading to false-negative results. Interestingly, several reports have documented measurable, serum CA 19-9 levels in Lewis-negative individuals with advanced pancreatic cancer, suggesting that alternative mechanisms may occasionally permit antigen expression in this subgroup (46). Notably, no universally accepted cutoff levels for MTC have been established to date, largely due to significant inter-assay variability and the absence of standardized prospective clinical studies. Studies in the literature differ in criteria used to define the reference population, study setup, and immunoassay used. Alencar *et al.* calculated a cutoff value of 18.3 U/mL (with a sensitivity of 83% and specificity of 91%) for the increase in CA 19-9 in advanced MTC following surgery using the Roche Elecsys assay (41). This cutoff is lower than the upper limit of normal of the assay and of CA 19-9 cutoff levels typically adopted with pancreatic cancer (approximately 37 U/mL).

### Clinical utility

Overall, although the prevalence of elevated serum CA 19-9 levels among patients with MTC is relatively low, elevated levels have been associated with adverse prognostic features in advanced disease. In recent years, growing evidence has suggested a potential prognostic role of CA 19-9 in advanced MTC. In two cases described by Milman *et al.* and Elisei *et al.*, metastatic MTCs showed markedly elevated serum CA 19-9 levels (47, 48). These results led to the hypothesis that an increased serum CA 19-9 reflects more aggressive disease behavior in advanced MTC. A prospective study involving 100 patients with MTC and nodal and/or distant metastases found that 16% had elevated serum CA 19-9 levels. This subgroup showed a significantly higher incidence of distant metastases and mortality compared to patients with normal CA 19-9 levels (49). Similar findings were reported by Alencar *et al.* and Lorusso *et al.*, who also observed that initial postoperative CEA levels were higher in those patients that showed disease progression and died from MTC compared to those that did not. They suggested that serum CA 19-9 levels and DT may complement CEA and should be considered in clinical decision-making, mainly when evaluating the appropriateness and the timing of initiating systemic therapy (41, 50).

## Pro-gastrin-releasing peptide (proGRP)

Pro-gastrin-releasing propeptide (proGRP) is a precursor of gastrin-releasing peptide (GRP). GRP is a bombesin-like neuropeptide hormone widely distributed in the central nerve system and in the gastrointestinal and pulmonary tract involved in tumor growth and differentiation. Since the measurement of GRP is difficult due to its short half-life, its precursor, proGRP, is currently determined and circulates in the form of three types of splicing variants with a half-life of 19–28 days (51). ProGRP is mainly used as a tumor marker of SCLC, but in the past decade, its potential role in MTC diagnosis and follow-up has emerged (52).

### Analytical and pre-analytical considerations

The first fully automated immunoassay dates back to 2009 (53). Subsequently, other fully automated immunometric immunoassays were developed, validated, and introduced in clinical settings. To date, no reference method or international standard has been established. Furthermore, there is still limited literature available on the analytical performance of different proGRP assays and comparisons between them (53, 54). Manual or semi-automated methods, such as enzyme-linked immunosorbent assays (ELISAs), can also be used, but mainly for research purpose. Pro-gastrin-releasing peptide measurement requires strict pre-analytical standardization due to its relative analytical lability. Blood should be collected in validated tubes, with consistent matrix use for longitudinal monitoring. Early literature data indicated that endogenous proteases, such as thrombin, generated during the coagulation process, can degrade proGRP in serum samples, suggesting the use of K_2/3_EDTA plasma for a reliable proGRP measurement (53). However, most current manufacturers offer optimized proGRP assays with minimized thrombin interference by using monoclonal antibodies targeting epitopes relatively resistant to endoproteolytic cleavage. As a result, both serum and plasma can nowadays be used for proGRP measurements (53, 54). Samples should be centrifuged and separated from cells within 1–2 h after collection. For short delays (≤24 h) before analysis, samples may be stored at 2–8°C; for longer storage, serum or plasma should be aliquoted and frozen (≤−20°C, preferably −70/−80°C), avoiding repeated freeze–thaw cycles. Transport must ensure appropriate temperature control. Samples with inadequate or unverifiable pre-analytical conditions should be considered non-conforming, and results should not be reported or must be clearly qualified, with repeat sampling under standardized conditions recommended. Elevated proGRP levels have been observed in patients with benign conditions, with circulating levels reaching and exceeding published reference ranges and cutoffs, especially in individuals with advanced renal failure (55). In addition, higher circulating levels of proGRP have been found in smokers and individuals with a high body mass index, and proGRP levels in adults tend to increase with age (56). Frequent causes of benign and malignant causes of serum pro-gastrin-releasing peptide (proGRP) elevation outside MTC are summarized in Table 7.

**Table 7:** Benign and malignant causes of serum pro-gastrin-releasing peptide (proGRP) elevation in conditions other than MTC (Molina *et al.* (56); Wojcik & Kulpa (62)).

Condition	Typical proGRP range (pg/mL)	Remarks
Benign conditions		
High body mass	60–150	Mechanism unknown
Renal insufficiency (CKD)	80–500	Correlates with eGFR
End-stage renal disease	200–1,000+	Marked elevation
Acute kidney injury	100–600	Often transient
Severe infections/sepsis	60–200	Non-specific; usually lower than SCLC
Chronic pulmonary disease	60–150	Mild elevation; overlap with smokers
Liver disease	60–150	Limited data; modest increase
Assay interference	Any	Suspect if clinical context is discordant
Malignant diseases		
Small cell lung cancer (SCLC)	200–10,000+	Primary indication; correlates with stage
Large cell neuroendocrine cancer (lung)	150–2,000	Lower than SCLC but often elevated
Neuroendocrine tumors (lung)	100–1,000	Typical/atypical carcinoids
GEP-neuroendocrine neoplasms	100–800	Variable secretion
Prostate neuroendocrine carcinoma	150–1,000	Usually advanced disease

MTC, medullary thyroid carcinoma.

### Reference ranges and cutoff levels

According to manufacturers’ instructions and the published literature, the upper reference limit for proGRP varies widely. Overall, since commercially available methods use different monoclonal antibodies, and neither a reference method nor a higher-level international standard has yet been established, reference intervals (and cutoffs) should be considered immunoassay-specific and, preferably, matrix-specific.

### Clinical utility

Currently available evidence does not allow for definitive conclusions regarding the utility of proGRP as a tumor marker in the diagnosis of MTC, and its diagnostic value remains uncertain (8, 31, 57). Published studies demonstrate a moderate sensitivity, limiting the use of proGRP in the screening and diagnosis of MTC, compared to CT and PCT, whose sensitivity is approximately 100% (52). In a recent international multicentric study, Schonebaum *et al.* demonstrated that proGRP, whether used alone or in combination with CT, does not provide additional diagnostic value for MTC in patients with thyroid nodules (31). On the other hand, proGRP has been shown to correlate with metastatic burden, with significantly elevated levels observed in patients with advanced MTC compared to those with localized disease (52). Post-operative reductions in proGRP levels further support its association with tumor load (58). Notably, in patients receiving treatment with tyrosine kinase inhibitors, longitudinal assessments of proGRP demonstrated a closer concordance with imaging responses than conventional biomarkers, such as CT or CEA, thereby highlighting its potential utility as an early biomarker of therapeutic efficacy or emerging resistance (52). All in all, the lack of assay standardization limits the ability to define standardized cutoff levels, which in turn hinders the integration of proGRP into established multi-marker diagnostic algorithms.

## Medullary thyroid biomarkers in fine-needle aspiration washouts

The measurement of CT in fine-needle aspiration washout (FNA-CT) proved to be a relevant diagnostic tool for suspected MTC and its metastases. While the cytopathological evaluation of thyroid nodules has a sensitivity rate of up to 65%, FNA-CT measurement demonstrates superior diagnostic accuracy, with sensitivity rates of 89–100% and specificities of 88.8–100% (8). The analytical performance varies depending on the immunoassay platform and cutoff levels used. Boi *et al.*, almost 20 years ago, reported 100% sensitivity and specificity using a chemiluminescence immunoassay with a 36 pg/mL cutoff in 36 lesions (59). Subsequently, consistency of results across different populations and assay platforms demonstrates the reliability of FNA-CT in clinical practice. A major advantage of FNA-CT measurement over conventional cytology is its independence from adequate cellular sampling, because CT is present in high levels both within MTC cells and in surrounding tissue, which allows reliable detection regardless of cellular content (60). This is particularly valuable in challenging cases involving cystic lesions, highly vascular nodules, or situations where patient-related factors limit optimal FNA cytology (61). Notably, proper implementation requires careful attention to pre-analytical and analytical considerations and thorough validation, of which evidence is often lacking in the literature. A sampling technique is crucial – specimens must truly represent the targeted lesion, whether thyroid nodules, thyroid bed areas, or suspicious lymph nodes. However, unlike the FNA cytology, it is possible to make a diagnosis using FNA-CT even though no epithelial cells were aspirated, since CT presents high levels both inside and in the neighboring area of the lesion. FNA washout calcitonin measurement requires a standardized and consistent operative protocol. In our clinical practice, we perform a dedicated FNA pass using a 22–25 G needle, which is rinsed twice with a fixed volume (1.0 mL) of 0.9% NaCl solution. Washout samples are thoroughly mixed and centrifuged at 2,000–3,000 *g* for 5–10 min, and calcitonin is measured in the supernatant. For short delays (≤24 h), samples are stored at −2–8°C, while a longer storage requires freezing (≤−20°C). Because calcitonin immunoassays are validated for serum/plasma, matrix effects are controlled by measuring analyte-free rinse-fluid blanks, which are required to yield values near zero. Samples obtained under non-validated conditions (i.e., FNA-CT) are clearly qualified. Laboratories must also implement protocols to detect potential interferences, including the hook effect, which can suppress CT measurements in cases with extremely high levels (8). A substantial interpretive challenge involves detectable FNA-CT levels in non-MTC conditions, caused by normal C-cells sampled during aspiration. This occurs more frequently in aspirates from middle and upper thyroid lobes, where C-cell density is naturally higher. Therefore, establishing appropriate cutoff levels becomes essential for maintaining diagnostic specificity while preserving sensitivity. These thresholds must be validated for each specific assay platform and adapted to local population characteristics, as inter-assay variability remains despite standardization efforts. Relatively recent research explored an alternative biomarker in FNA washouts, proGRP. Liang *et al.* evaluated FNA-proGRP measurement in 235 thyroid nodules with different histological categories, MTC, and non-MTC. Their findings showed significantly elevated FNA-proGRP levels in MTC nodules (median 2,096 pg/mL) compared to other thyroid pathologies. Using a cutoff of 22.77 pg/mL, they achieved a sensitivity of 94.12%, a specificity of 98%, and strong statistical agreement in differentiating MTC from non-MTC thyroid nodules (57). These results are promising but require further validation. In conclusion, FNA-CT measurement is a clinically validated approach that significantly improves diagnostic accuracy compared to cytology alone to confirm the medullary origin of thyroid nodules showing serum CT elevations in the gray zone. However, this approach may become less relevant, as recent evidence demonstrates that serum PCT provides excellent diagnostic accuracy in ruling out MTC in such patients. In contrast, CT measurement in FNA washouts remains pivotal for evaluating suspicious cervical lymph nodes in patients with an established diagnosis of MTC, either before surgery or during postoperative surveillance, as it supports the localization of metastatic disease and surgical planning.

## Conclusions

**Figure 1:**
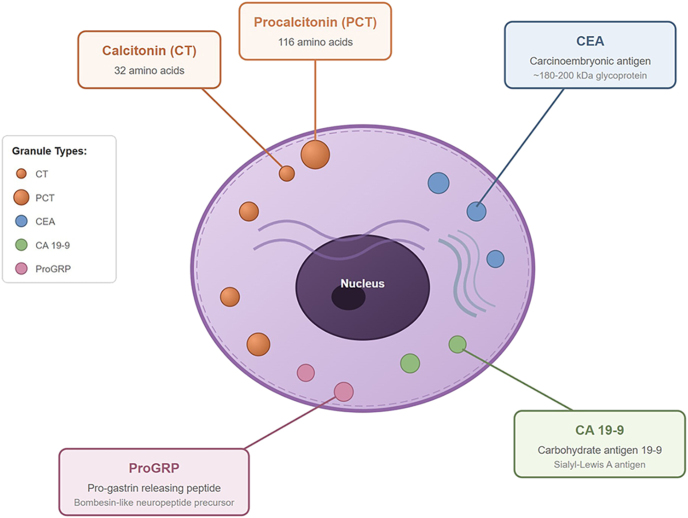
Circulating biomarkers used in MTC.

MTC is characterized by the secretion of multiple peptide biomarkers that, when correctly measured and interpreted, provide essential information for diagnosis, prognosis, and follow-up. A schematic representation of the main biomarkers applied in MTC diagnosis and follow-up is reported in Fig. 1. Serum (blood) biomarkers include calcitonin, procalcitonin, carcinoembryonic antigen (CEA), CA 19-9, and pro-gastrin-releasing peptide (proGRP). Fine-needle aspiration (FNA) washout fluid can also be analyzed for calcitonin and proGRP to improve lesion characterization and diagnostic accuracy. The laboratory thus represents a cornerstone of MTC management, supporting both diagnostic confirmation and clinical decision-making throughout the disease course. However, the effective use of MTC biomarkers is still challenged by analytical variability, biological interferences, and the lack of universal interpretative standards. Addressing these limitations requires ongoing assay harmonization, external quality assessment programs, and multidisciplinary dialogue between clinical chemists and treating physicians. To optimize patient care, clinical pathologists and clinical chemists should actively participate in multidisciplinary tumor boards and case discussions, where their expertise in assay performance, interferences, and result interpretation can guide appropriate diagnostic and therapeutic decisions. Such integrated collaboration is pivotal to resolving laboratory-related ambiguities, ensuring reliable biomarker interpretation, and ultimately improving outcomes in patients with MTC.

## Declaration of interest

The authors declare that there is no conflict of interest that could be perceived as prejudicing the impartiality of the work reported.

## Funding

This work did not receive any specific grant from any funding agency in the public, commercial, or not-for-profit sector.
